# Associations Between Dietary Patterns, Nutrient Intake, and Serum Biomarkers in Community-Dwelling Older Adults in Northern Thailand: A Cross-Sectional Study

**DOI:** 10.3390/nu18132204

**Published:** 2026-07-07

**Authors:** Minatsu Kobayashi, Nobuko Shimizu, Noboru Hasegawa, Takako Yamada, Tomohiro Umemura, Mayumi Kato, Kyosuke Yorozuya, Piyathorn Rengrew, Pattaranai Chaiprom, Patana Nakatong, Kamolthip Thipsungwan, Hunsa Sethabouppha, Nattaya Suwankruhasn, Chalinee Suvanayos

**Affiliations:** 1 Department of Food Science, Faculty of Home Economics, Otsuma Women’s University, 12, Sanban-cho, Chiyoda-ku, Tokyo 102-8357, Japan; 2 Department of Community Health Nursing, Faculty of Nursing, Toyama Prefectural University, 2-2-78 Nishinagae, Toyama 930-0975, Japan; n-shimizu@pu-toyama.ac.jp; 3 Department of Rehabilitation, Faculty of Rehabilitation, AICHI Medical College of Rehabilitation, 519 Ichiba, Kiyosu 452-0931, Aichi, Japan; nhasegaw@yuai.ac.jp (N.H.); mayumi@yuai.ac.jp (M.K.); 4 Department of Occupational Therapy, Faculty of Health Science, Bukkyo University, Higashitoganoo-cho, Nishinokyo, Nakagyo-ku, Kyoto 604-8418, Japan; tayamada@bukkyo-u.ac.jp (T.Y.); yorozuya.kyosuke@gmail.com (K.Y.); 5 Department of Sports and Health Sciences, School of Health and Sport Sciences, Chukyo University, 101 Tokodachi, Kaizu-cho, Toyota 470-0393, Japan; ume-tomo@sass.chukyo-u.ac.jp; 6 Faculty of Nursing, Lampang Rajabhat University, 119 Lampang-Mae Ta Road, Chomphu Subdistrict, Mueang District, Lampang 52100, Thailand; piyathorn@g.lpru.ac.th (P.R.); patananak@g.lpru.ac.th (P.N.); kamolthip@g.lpru.ac.th (K.T.); 7 Department of Community Public Health, Faculty of Science, Lampang Rajabhat University, 119 Lampang-Mae Ta Road, Chomphu Subdistrict, Mueang District, Lampang 52100, Thailand; pattaranai@g.lpru.ac.th; 8 Faculty of Nursing, Chiang Mai University, 110/406 Inthawaroros Road, Suthep, Muang, Chiang Mai 50200, Thailand; hunsa.s@cmu.ac.th (H.S.); nattaya.s@cmu.ac.th (N.S.); chalinee.s@cmu.ac.th (C.S.)

**Keywords:** dietary patterns, nutritional epidemiology, older adults, Thailand, nutrient intake, serum biomarkers, principal component analysis

## Abstract

**Background/Objectives**: This study aimed to identify dietary patterns among community-dwelling older adults in northern Thailand and examine their associations with nutrient intake, serum biomarkers, and anthropometric indicators. **Methods**: A cross-sectional study was conducted among 117 older adults in Lampang and Chiang Mai provinces in Northern Thailand. Dietary intake was assessed using a semiquantitative food frequency questionnaire. Dietary patterns were derived using principal component analysis, and participants were classified into mutually exclusive dietary pattern groups based on their highest factor scores, which was defined as the dominant pattern. Differences in nutrient intake were evaluated using the Kruskal–Wallis test. Serum biomarkers and anthropometric indicators were assessed using an analysis of covariance adjusted for age, sex, residential area, total energy intake, and body mass index. **Results**: Four dietary patterns were identified: diverse traditional, processed staple-based, tropical fruit, and a Western diet high in fats and sweets. Dietary fiber intake differed significantly among the four patterns, with the highest level in the diverse traditional and lowest in the processed staple patterns. No significant differences were observed in total energy or major nutrient intake. Total cholesterol levels differed significantly, with lower levels in the traditional diet pattern than in the tropical fruit and Western diet patterns. **Conclusions**: Dietary patterns among older adults in northern Thailand may be associated with dietary fiber intake and serum total cholesterol levels. A traditional diet rich in vegetables and fish may be linked to more favorable nutrient intake and lipid profiles.

## 1. Introduction

Rapid population aging across Southeast Asian countries, including Thailand, has made maintaining health and prolonged independent living among older adults an important public health issue [1]. Nutritional status in older age is closely associated with the maintenance of quality of life and prevention of lifestyle-related diseases, such as cardiovascular disease, diabetes, and osteoporosis [2]. However, older adults are particularly vulnerable to both inadequate and excessive nutrient intake owing to age-related physiological changes, chronic comorbidities, reduced appetite, and diminished chewing and swallowing functions. In particular, insufficient intake of nutrients such as vitamin D and dietary fiber as well as excessive intake of fat and carbohydrates have been highlighted as key nutritional concerns in older populations [3,4].

Recent nutritional epidemiology has shifted focus from analyzing single nutrients or food groups to evaluating holistic dietary patterns [5]. Principal component analysis (PCA) or factor analysis helps identify actual dietary habits by capturing combinations of foods consumed together; this enables a comprehensive evaluation of the relationships between culturally specific eating behaviors and health indicators [6]. This dietary pattern approach is particularly useful for understanding the relationship between dietary behavior and health in regions undergoing rapid dietary transitions. In Thailand, diets have transitioned from traditional patterns centered on rice, vegetables, and fish to those that include processed and high-fat foods, leading to increased diversity in older adults’ diets [7]. In northern Thailand, while traditional dietary culture has been relatively well preserved, it has also been influenced by urbanization and changes in the food environment, making it important to characterize the dietary habits of older adults. Such changes in dietary practices may affect health later in life; therefore, it is important to clarify the associations between dietary patterns that reflect the actual dietary composition and health indicators in older populations.

However, studies on dietary patterns among older adults in Thailand remain limited, and most existing research has focused on specific nutrients or food groups [8]. Furthermore, few studies have simultaneously examined the characteristics of nutrient intake across dietary patterns and their associations with objective biomarkers such as serum vitamin D and lipid-related indicators, particularly among community-dwelling older adults in northern Thailand [9]. Moreover, evidence remains limited regarding the influences of regional dietary diversity and local food environments. To address this, we focused on older adults residing in the Chiang Mai and Lampang provinces in Northern Thailand. Using PCA, we aimed to identify dominant dietary patterns and their associations with nutrient intake, serum biomarkers, and anthropometric indicators. This geographically specific approach enabled us to examine region-specific dietary patterns despite the relatively small sample size.

## 2. Methods

### 2.1. Study Design and Participants

This cross-sectional study included community-dwelling older adults residing in Lampang and Chiang Mai provinces in northern Thailand. The participants were recruited between 2023 and 2024.

Participants from Lampang Province were recruited based on recommendations from the Subdistrict Health Promotion Hospital directors, and 20 participants each were drawn from three communities: Ban Tha Tok (Thung Fai Subdistrict, Mueang Lampang District), Ban Kluai Phae (Kluai Phae Subdistrict, Mueang Lampang District), and Ban Hong Ha (Nam Cho Subdistrict, Mae Tha District). Overall, 66.7% of participants were from urban/peri-urban areas (Mueang Lampang District), whereas 33.3% were from rural areas (Mae Tha District), reflecting a community-based sample with both urban and rural representation.

Participants in Chiang Mai province were recruited via the heads of older adult community groups, originally established through a community outreach initiative by Chiang Mai University. In addition, purposive sampling was used to recruit participants from both urban and peri-urban areas through public announcements and invitation posters distributed in senior clubs affiliated with the Faculty of Nursing, Chiang Mai University. The sample comprised 58 participants from Chiang Mai, Thailand, with 51.7% residing in urban (all from Mueang District) and 48.3% in rural areas (from six districts: Sansai and Hangdong [25.0% each]; Mae Wang, Sankamphaeng, and Saraphi [14.3% each], and Mae Rim [7.1%]). This distribution reflects a homogeneous urban sample and a geographically diverse rural sample largely characterized by peri-urban areas.

Eligible participants were aged ≥60 years, had no severe physical illness, lived independently, communicated fluently in Thai, and consented to participate. Individuals with psychiatric symptoms were excluded. The participants were allowed to withdraw from the study at any time if they experienced discomfort or emotional distress.

Individuals were selected based on pre-screening blood test results to ensure they lacked severe health conditions that could interfere with the implementation or evaluation of the study. Written informed consent was obtained from all participants before enrollment after fully explaining the study objectives and procedures.

Eligibility was further confirmed based on self-reported health status, documented ability to complete the study procedures, and structured interviews by trained research staff. Individuals with psychiatric symptoms were identified through self-reports or interviews and excluded, and final eligibility was determined by the research team.

A total of 58 participants from Chiang Mai province and 60 from Lampang province were enrolled. After excluding one participant owing to missing blood data, 117 participants were included in the final analysis. The sample should be interpreted as a community-based convenience sample and may not be fully representative of older adults in northern Thailand.

Ethical approval of the study design was obtained from the Central Research Ethics Committee (CREC) in Thailand (approval number: COA-CREC 062/2019, approved on 31 October 2019) and the Human Research Ethics Committee of Boromarajonani College of Nursing, Lampang (certificate number: E2567-055, approved on 3 September 2024).

### 2.2. Data Collection Period and Variables

Data collection was conducted at different times in each region: in 2023 in Chiang Mai Province and in 2024 in Lampang Province. In both regions, dietary assessments, questionnaire surveys, anthropometric measurements, and blood tests were performed concurrently, ensuring all data were collected at the same time point for each participant.

(1) Demographic Characteristics

Data on age, sex, residential area (Lampang or Chiang Mai province), education level, and living arrangements were collected using a structured, self-administered questionnaire conducted at the time of the survey.

(2) Dietary Assessment and Nutrient Intake

Dietary intake was assessed using a semiquantitative food frequency questionnaire (SFFQ). The SFFQ was adapted from an existing FFQ developed for Thai adults and modified to include food and mixed dishes commonly consumed by older adults in Chiang Mai and Lampang. The SFFQ included 128 food items that were subsequently grouped into 48 food groups by combining similar foods. Portion sizes were estimated using the standard portion sizes commonly applied in Thai dietary surveys, although these were not specifically validated for older adults. Nutrient intake was calculated using INMUCAL-Nutrients Version 4.0 (Institute of Nutrition, Mahidol University, Thailand) [10]. The FFQ was structured to collect information on the frequency and standard portion size of each food and beverage item, and individual nutrient intake was estimated based on the responses. The existing Thai SFFQ has been reported to be useful for estimating energy and major nutrient intake [11]. Using the food intake frequency data, the intakes of energy, protein, fat, carbohydrates, dietary fiber, cholesterol, vitamin D, and sodium were calculated. The reported energy intake ranged from 315 to 8400 kcal/day. None of the participants were excluded because of implausible energy intake.

(3) Anthropometric Measurements

Trained medical and research staff performed anthropometric measurements at each study site. Height and body weight were measured with participants wearing light clothing and no shoes and recorded to the nearest 0.1 cm and 0.1 kg, respectively. Body mass index (BMI; kg/m^2^) was calculated as weight (kg) divided by height (m) squared. Body fat percentage (%) was measured using bioelectrical impedance analyzer (ACCUNIQ BC300, SELVAS Healthcare Inc., Daejeon, Republic of Korea). All the anthropometric measurements were performed on the same day for each participant.

(4) Blood Tests

Blood tests were conducted on the day of the survey, using fasting blood samples. Medical staff collected blood samples in each study area. Samples from Chiang Mai were analyzed at the AMS-Clinical Service Center, Faculty of Associated Medical Sciences, Chiang Mai University (110 Inthawarorot Rd., Sriphum, Muang, Chiang Mai 50200, Thailand), whereas samples from Lampang were analyzed at MP Lab Medical Technology Clinic (Lampang, Thailand) using standard laboratory methods. Serum 25-hydroxyvitamin D (25(OH)D) was analyzed at Bangkok R.I.A. Lab Co., Ltd., Chiang Mai Branch (Chiang Mai, Thailand). Measured parameters included 25(OH)D, total cholesterol, low-density lipoprotein cholesterol, high-density lipoprotein cholesterol, triglycerides, and fasting blood glucose. Blood test results were obtained from each facility and registered in an existing database for use in this analysis.

(5) Lifestyle-Related Variables

Smoking status was assessed using a questionnaire and included in the analysis. Information on physical activity and sun exposure was excluded from the statistical analyses because of missing data in a subset of participants to ensure consistency of the analysis.

### 2.3. Dietary Pattern Derivation

Dietary patterns were derived using food intake data obtained from the SFFQ based on the intake frequency aggregated by food groups. Foods and dishes were classified into 48 food groups according to their nutritional characteristics and similarities in dietary habits, and the intake frequency was calculated for each group. Prior to PCA, the food group intake frequency data were standardized (z-scores) using the mean and standard deviation. PCA was then conducted using the standardized food group intake frequency data to extract dietary patterns [5,6]. Varimax rotation with Kaiser normalization (orthogonal rotation) was applied to the factor solution. The number of factors to be retained was determined by considering the eigenvalues, scree plot, and interpretability of the factors; four factors were retained in this study. Food groups characterizing each factor were identified based on factor loadings, and those with absolute factor loadings of ≥0.30 were considered representative of the respective factor. Each dietary pattern was labeled according to the composition of the food groups. The eigenvalues, explained variances, and cumulative variances of each factor are listed in Table 1. Factor scores for each participant were calculated using regression. The suitability of PCA was assessed using the Kaiser–Meyer–Olkin (KMO) measure and Bartlett’s test of sphericity.

### 2.4. Statistical Analysis

Participants were classified into four groups based on the dietary pattern with the highest factor score, defined as the dominant dietary pattern, and factor scores were used as continuous and categorical variables. As participant characteristics and nutrient intake were not normally distributed, continuous variables are presented as medians (25th–75th percentiles). Differences in age, BMI, body fat percentage, and nutrient intake among the dominant dietary pattern groups were assessed using the Kruskal–Wallis test, followed by post hoc multiple comparisons using the Bonferroni method when significant differences were observed. Differences in categorical variables (sex, residential area, alcohol consumption, and educational level, and presented as *n* [%]) were examined using the chi-square test or Fisher’s exact test, and post hoc comparisons were conducted using adjusted residuals when significant differences were found.

For serum biomarkers and anthropometric indicators, adjusted means and 95% confidence intervals were estimated using analysis of covariance (ANCOVA/GLM), adjusted for age, sex, residential area, total energy intake, and BMI. Before analysis, model assumptions, including the normality of residuals and homogeneity of variance, were assessed. Logarithmic transformation was applied as necessary to meet the model assumptions, and the results are presented as back-transformed values. BMI was included as a covariate to account for differences in body composition, which may influence serum biomarker levels.

As a supplementary analysis, a multiple linear regression analysis was conducted using dietary pattern factor scores as continuous variables and serum biomarkers as dependent variables. The β coefficients represent the change in outcomes associated with a one-standard-deviation increase in dietary pattern scores, adjusted for age, sex, residential area, BMI, and total energy intake.

All statistical analyses were performed using SPSS Statistics version 29 (IBM Corp., Armonk, NY, USA). All tests were two-sided, and a *p*-value < 0.05 was considered statistically significant.

## 3. Results

Before conducting the PCA, the dataset was assessed for factorability. The KMO measure of sampling adequacy was 0.712, and Bartlett’s test of sphericity was significant (*p* < 0.001), confirming the data were suitable for factor analysis.

PCA of the food group intake frequency data identified the following four dietary patterns based on the factor loadings of the major food groups (Table 1): diverse traditional (P1), processed staple (P2), tropical fruit (P3), and Western diet high in fats and sweets (P4). Factor scores were calculated for each participant, and the pattern with the highest factor score was defined as the dominant dietary pattern.

Table 2 presents the participants’ characteristics according to their dominant dietary patterns. The study sample was predominantly female, with 107 of the 117 participants being women. The median age of the 117 participants was 66 years (25th–75th percentile, 63–70 years). Age did not differ significantly among dietary patterns (*p* = 0.161), whereas BMI differed significantly (*p* = 0.005). Post hoc comparisons indicated a significantly higher BMI in individuals with a dominant Western diet high in fats and sweets pattern (P4) than in those with a dominant tropical fruit pattern (P3). Body fat percentage did not differ significantly among dietary patterns (*p* = 0.231). The distribution of residential areas differed significantly among dietary patterns (*p* = 0.043). A higher number of participants from Lampang Province favored the processed staple pattern (P2), whereas a higher number of participants from Chiang Mai Province favored the Western diet high in fats and sweets pattern (P4). No significant differences were observed among dietary patterns in terms of sex, alcohol consumption, or educational level.

Table 3 shows nutrient intakes according to the dominant dietary patterns. Nutrient intakes are presented as medians (25th–75th percentiles). Group comparisons revealed a significant difference in dietary fiber intake among the dietary patterns (*p* = 0.032). Dietary fiber intake was relatively higher in the diverse traditional pattern (P1) and relatively lower in the processed staple pattern (P2). In contrast, no significant differences were observed among the dietary patterns in total energy, protein, fat, carbohydrate, vitamin D, or sodium intake.

Table 4 presents the adjusted means (95% confidence intervals) of serum biomarkers and anthropometric indicators according to dominant dietary patterns. Differences among the groups were assessed using ANCOVA. Total cholesterol levels differed significantly among dietary patterns (*p* = 0.006). Post hoc Bonferroni tests indicated that the total cholesterol levels were significantly lower in individuals with a dominant diverse traditional pattern (P1) than in those with a dominant tropical fruit pattern (P3) and Western diet high in fats and sweets pattern (P4). In contrast, no statistically significant differences were observed in the dietary patterns in serum vitamin D (*p* = 0.074), triglycerides (*p* = 0.091), blood glucose (*p* = 0.174), BMI (*p* = 0.109), or body fat percentage (*p* = 0.083).

In a supplementary analysis, we analyzed the dietary pattern factor scores as continuous variables using multiple regression analysis (Table S1). Although some associations with serum biomarkers were observed, the overall findings were consistent with those of the primary analysis. Additional analyses using continuous factor scores showed similar trends, with higher scores for the diverse traditional pattern being associated with higher dietary fiber intake and lower total cholesterol levels.

## 4. Discussion

In this study of community-dwelling older adults in northern Thailand, four dietary patterns were identified: diverse traditional, processed staple, tropical fruit, and Western diet high in fats and sweets. Differences in dietary patterns may be associated with variations in dietary fiber intake and total cholesterol levels.

The diverse traditional dietary pattern, centered on rice as a staple food, along with vegetables, fish, and traditional mixed dishes, is considered similar to the traditional Thai diet. In this study, this dietary pattern showed a relatively higher dietary fiber intake; this finding is consistent with a previous study among older adults in Southeast Asia, which reported traditional diets characterized by a relatively higher intake of vegetables and plant-based foods [12]. In contrast, the processed staple pattern was characterized by a relatively higher consumption of refined staple foods and processed foods, and a tendency toward lower dietary fiber intake. Although differences in dietary fiber intake were observed among the dietary patterns, no significant differences were found in total energy or major nutrient intake. In older adults, total energy intake tends to be relatively stable and dietary patterns are often centered on staple foods; this may make differences in individual nutrient intake less apparent [6]. Furthermore, dietary pattern analysis reflects food combinations and overall consumption tendencies and does not necessarily result in large differences in individual nutrient intake [5]. Although some food items showed cross-loadings across multiple dietary patterns (e.g., durian and vermicelli), each pattern was labeled based on the overall combination of food groups with the highest factor loadings and their dominant dietary characteristics. Therefore, the pattern names reflect general dietary tendencies rather than individual food items. The identified patterns were also consistent with commonly observed dietary behaviors in the study population, reflecting both the nutritional characteristics and local food culture.

In this study, dietary patterns were found to be associated with serum lipid profiles, with the diverse traditional pattern associated with lower total cholesterol levels compared with the other patterns. However, no significant differences were observed in the other lipid parameters. Nevertheless, our results are generally in line with the previous observations that diets rich in vegetables and fish have beneficial effects on lipid metabolism and cardiovascular disease risk [13,14]. Similar trends were observed in the supplementary analyses using the continuous factor scores. However, given the cross-sectional study design, causal relationships could not be established, and lipid levels may be influenced by various factors, including medication use, comorbidities, body composition, socioeconomic status, and other lifestyle factors. Therefore, the findings should be interpreted with caution. These differences may reflect regional food culture, food availability, and the degree of urbanization. In contrast, no significant differences in triglyceride, blood glucose, or serum vitamin D levels were observed among the dietary patterns. In particular, serum vitamin D is known to be influenced not only by dietary intake but also by multiple factors, including cutaneous synthesis through ultraviolet exposure, physical activity, and the duration of sun exposure [15]. Consequently, a direct association between dietary patterns and serum vitamin D may not be clearly evident. In this study, serum vitamin D levels did not significantly differ among dietary patterns, which may be attributable to the influence of sun exposure and lifestyle factors. Therefore, evaluating vitamin D status requires a holistic approach that considers not only dietary intake but also lifestyle factors such as physical activity and sun exposure.

In addition, a relatively higher BMI was observed in the group consuming a Western diet high in fats and sweets. Such diets are energy-dense and may be associated with weight gain [16] as well as an increased risk of obesity and metabolic syndrome [17]. Interestingly, differences in the distribution of dietary patterns were observed according to residential area, with a higher proportion of participants from Lampang Province following the processed staple pattern and a higher proportion from Chiang Mai Province following the Western diets high in fats and sweets pattern. These differences may reflect regional food culture, food availability, and the degree of urbanization. Chiang Mai is a more urbanized area with greater access to diverse and processed foods, whereas Lampang is relatively less urbanized, where traditional dietary practices and locally available foods may play a larger role. Such differences in urbanization, food availability, and local food culture may have contributed to the variation in dietary patterns observed in this study.

The strength of this study is that dietary pattern analysis was used to evaluate overall habitual dietary behavior while simultaneously examining nutrient intake, anthropometric measurements, and serum biomarkers. In particular, studies simultaneously assessing dietary patterns and objective health indicators among community-dwelling older adults in Thailand are limited [18]. In this study, multiple dietary patterns were identified among community-dwelling older adults in northern Thailand, which may be associated with differences in dietary fiber intake and serum total cholesterol levels.

However, this study has several limitations. First, as this was a cross-sectional study, causal relationships between dietary patterns and health indicators could not be established [19]. Second, the sample size was relatively small and limited to two regions in northern Thailand, which may limit the stability of the identified dietary patterns. In particular, in PCA, the subject-to-variable ratio is known to directly influence the stability and reproducibility of the extracted dietary patterns [20,21]. Therefore, caution is required when generalizing the findings. Moreover, the use of non-probabilistic, community-based sampling approach, wherein the sample was interpreted as a community-based convenience sample, may further limit the generalizability of the findings. Furthermore, the study population was predominantly female, which may limit the generalizability of the findings to older men. In addition, classifying participants by their dominant dietary pattern—determined by the highest factor score—ignores the overlapping nature of PCA patterns. However, dietary patterns derived by PCA are not mutually exclusive, and this approach may lead to a loss of information compared to analyses using continuous factor scores. Finally, because BMI may lie in the causal pathway between dietary patterns and metabolic outcomes, including it as a covariate in the lipid biomarker analysis introduces the risk of overadjustment. However, in this study, PCA was conducted after aggregating food groups, and dietary patterns were determined by comprehensively considering eigenvalues, the scree plot, and the interpretability of the factors [5,6]. Third, dietary intake was assessed using the SFFQ, and measurement errors may have occurred [22]. Moreover, a wide range of reported energy intakes was observed (315–8400 kcal/day), and implausible values were not excluded. Sensitivity analyses excluding the upper and lower 1% of energy intake yielded similar results. Given the relatively small sample size, all the participants were included in the main analysis. These factors may have introduced measurement errors, and the findings should be interpreted with caution. Furthermore, residual confounding may remain as important variables such as physical activity and sun exposure were not assessed in this study. Finally, data collection was conducted at different times in each region (Chiang Mai in 2023 and Lampang in 2024), which may have introduced heterogeneity due to seasonal and temporal variations in food availability and consumption patterns. Regional differences, including the more urbanized setting of Chiang Mai compared to Lampang, may also have contributed to the variation in dietary patterns observed in this study. Future large-scale and longitudinal studies are needed to further investigate the association between dietary patterns and health in older adults.

## 5. Conclusions

This study identified four dietary patterns among community-dwelling older adults in northern Thailand and revealed significant associations between these patterns, daily dietary fiber intake, and serum total cholesterol levels.

These findings provide preliminary evidence of dietary pattern variations among community-dwelling older adults in northern Thailand and may inform future large-scale studies and culturally tailored nutrition research. However, given the cross-sectional design and exploratory nature of the analysis, further studies are required to confirm these findings.

## Figures and Tables

**Table 1:** Factor loadings and variance explained for four dietary patterns among older Thai adults.

Food Item	P1 Diverse Traditional Pattern	P2 Processed Staple Pattern	P3 Tropical Fruit Pattern	P4 Western Diet High in Fats and Sweets Pattern
Onions	0.794		0.317	
Carrots	0.787			
Shallot	0.781			
Bean sprouts	0.743			
Morning glory	0.729			
Mushrooms	0.720			
Coriander	0.672			
Bell peppers	0.659			
Pumpkin	0.627			
Broccoli	0.588		0.429	
Beef Sirloin	0.569			
Cabbage	0.481	0.331		
Tofu	0.432			
Shrimp	0.366			
Dried fish		0.877		
Pork sausage		0.762		
Durian		0.740		0.331
Vermicelli		0.738		0.359
Yogurt		0.571		
Macaroni		0.490		
Desserts with coconut milk		0.434		
White Bread		0.359		
Ripe papaya			0.835	
Banana	0.361		0.755	
Citrus fruits			0.736	
Pineapple	0.350		0.703	
Pear		0.374	0.661	
Rambutan	0.375		0.661	
Pizzas				0.841
Fried potatoes				0.800
Eggs				0.635
Ice cream				0.592
Potato				0.582
Chicken breast				0.442
Milk				0.434
Eigenvalue	6.9	4.3	4.1	3.9
Variance explained	14.3	9	8.4	8.1
Cumulative variance	14.3	23.3	31.7	39.8

Note: Dietary patterns were derived using principal component analysis with varimax rotation and Kaiser normalization. Only factor loadings ≥ 0.30 are shown.

**Table 2:** Characteristics of participants according to the dominant dietary pattern.

	All (*n* = 117)	P1 (*n* = 21)	P2 (*n* = 26)	P3 (*n* = 39)	P4 (*n* = 31)	*p*-Value	Post Hoc
Age (years)	66 (63–70)	68 (64–72)	66 (63–70)	66 (63–71)	65 (63–67)	0.161	
BMI (kg/m^2^)	23.6 (21.1–25.5)	22.9 (20.7–25.1)	23.7 (22.1–25.3)	21.5 (19.7–24.7)	24.2 (23.1–26.5)	0.005	P3 vs. P4
Body fat (%)	32.9 (28.9–36.5)	34.0 (28.5–36.4)	31.9 (29.8–36.4)	31.7 (28.4–35.2)	34.1 (32.0–38.1)	0.231	
Sex, *n* (%)							
Male	10 (8.5)	2 (9.5)	2 (7.7)	1 (2.6)	5 (16.1)	0.249	
Female	107 (91.5)	19 (90.5)	24 (92.3)	38 (97.4)	26 (83.9)		
Residential Area, *n* (%)							
Chiang Mai	57 (48.7)	11 (52.4)	7 (26.9)	19 (48.7)	20 (64.5)	0.043	P2 vs. P4
Lampang	60 (51.3)	10 (47.6)	19 (73.1)	20 (51.3)	11 (35.5)		
Alcohol drinking, *n* (%)							
Yes	32 (27.4)	5 (23.8)	9 (34.6)	11 (28.2)	7 (22.6)	0.755	
No	85 (72.6)	16 (76.2)	17 (65.4)	28 (71.8)	24 (77.4)		
Education level, *n* (%)							
Up to middle school graduation	37 (31.6)	7 (33.3)	8 (30.8)	13 (33.3)	9 (29.0)	0.980	
High school graduation or above	80 (68.4)	14 (66.7)	18 (69.2)	26 (66.7)	22 (71.0)		

Note: Continuous variables are presented as medians (25th–75th percentiles), and categorical variables are presented as *n* (%). Differences among groups were assessed using the Kruskal–Wallis test with Bonferroni-adjusted pairwise comparisons. Categorical variables were analyzed using the chi-square test or Fisher’s exact test. Post hoc comparisons were conducted using adjusted standardized residuals (|residual| ≥ 1.96). Dietary patterns were classified based on the highest factor score as follows: P1, diverse traditional pattern; P2, processed staple pattern; P3, tropical fruit pattern; and P4, Western diet high in fats and sweets pattern.

**Table 3:** Nutrient intake according to the dominant dietary pattern.

	All (*n* = 117)	P1 (*n* = 21)	P2 (*n* = 26)	P3 (*n* = 39)	P4 (*n* = 31)	*p*-Value	Post Hoc
Energy (kcal/day)	2021 (1257–3179)	1884 (1186–3426)	1944 (1280–3063)	2075 (1255–3276)	2042 (1273–2495)	0.968	
Protein (g/day)	73.1 (39.7–129.9)	91.9 (52.2–154.2)	69.3 (35.4–113.3)	93.4 (39.5–139.5)	68.3 (38.9–115.2)	0.734	
Fat (g/day)	48.2 (21.9–89.5)	52.8 (25.2–127.1)	39.1 (20.5–100.8)	55.1 (16.9–88.9)	48.5 (21.5–69.6)	0.898	
Cholesterol (mg/day)	300.6 (169–620)	358.5 (222–980)	266.2 (116–668)	293.5 (147–586)	304.2 (162–547)	0.511	
Carbohydrate (g/day)	312.3 (205.2–485.0)	293.4 (179.8–485.0)	296.5 (225.3–465.8)	328.5 (147.3–598.9)	320.8 (235.6–442.7)	0.892	
Dietary Fiber (g/day)	20.6 (9.1–46.9)	37.9 (15.7–62.6)	12.4 (6.2–28.2)	20.6 (7.9–52.6)	18.5 (7.4–42.1)	0.032	P1 vs. P2
Vitamin D (µg/day)	4.3 (2.8–11.2)	8.3 (3.8–15.0)	3.7 (2.0–11.0)	4.1 (2.9–11.3)	4.6 (2.9–8.2)	0.142	
Sodium (g/day)	1.8 (0.9–5.2)	2.9 (1.3–7.5)	1.7 (1.0–4.8)	1.4 (0.8–7.6)	1.9 (0.8–4.3)	0.742	

Note: Continuous variables are presented as medians (25th to 75th percentiles). Differences among groups were assessed using the Kruskal–Wallis test with Bonferroni-adjusted pairwise comparisons. Dietary patterns were classified based on the highest factor score as follows: P1, diverse traditional pattern; P2, processed staple pattern; P3, tropical fruit pattern; and P4, Western diet high in fats and sweets pattern.

**Table 4:** Adjusted means (95% CI) of serum biomarkers by dominant dietary pattern.

Biomarker	P1 (*n* = 21)	P2 (*n* = 26)	P3 (*n* = 39)	P4 (*n* = 31)	*p*-Value	Post Hoc
Serum vitamin D (ng/mL)	24.7 (21.9–27.8)	22.8 (20.5–25.4)	27.4 (25.1–30.0)	24.4 (22.0–27.0)	0.074	
Total cholesterol (mg/dL)	185.9 (169.4–204.2)	201.7 (185.7–219.0)	220.7 (205.6–236.7)	227.0 (209.3–245.9)	0.006	P1 vs. P3, P1 vs. P4
Triglycerides (mg/dL)	93.2 (77.3–112.4)	105.7 (89.7–124.8)	102.8 (89.4–118.4)	127.4 (108.4–149.6)	0.091	
Blood glucose (mg/dL)	103.9 (96.0–112.3)	101.3 (94.3–108.7)	101.3 (95.5–107.4)	93.3 (87.3–99.8)	0.174	
BMI (kg/m^2^)	23.4 (21.8–25.0)	24.0 (22.6–25.5)	22.3 (21.2–23.5)	24.4 (23.1–25.8)	0.109	
Body Fat (%)	32.9 (30.4–35.5)	34.2 (31.9–36.6)	30.8 (29.1–32.6)	33.7 (31.6–35.9)	0.083	

Note: Values are presented as adjusted means (95% confidence interval). Differences between groups were assessed using analysis of covariance (ANCOVA; Type III test) with Bonferroni-adjusted pairwise comparisons. Values were log-transformed for analysis and back-transformed for presentation where appropriate. Models were adjusted for age, sex, residential area, and total energy intake, and BMI was additionally adjusted for all outcomes, except BMI and body fat percentage. Dietary patterns were classified based on the highest factor score as follows: P1, diverse traditional pattern; P2, processed staple pattern; P3, tropical fruit pattern; P4, Western diet high in fats and sweets pattern.

## Data Availability

The data presented in this study are not publicly available because of privacy and ethical restrictions but are available from the corresponding author upon reasonable request, subject to approval by the relevant ethics committees.

## Supplementary Materials

The following supporting information can be downloaded at: https://www.mdpi.com/article/10.3390/nu18132204/s1, Table S1: Association between dietary pattern scores and serum biomarkers.

## Abbreviations

Principal component analysis (PCA), body mass index (BMI); semi-quantitative food frequency questionnaire (SFFQ); confidence interval (CI); analysis of covariance (ANCOVA); standard deviation (SD).
